# Clinical evaluation of EUCAST rapid antimicrobial susceptibility testing in Gram-negative bacilli bloodstream infections: a real-world retrospective study

**DOI:** 10.3389/fmicb.2026.1874736

**Published:** 2026-07-13

**Authors:** Peng-Peng Tian, Hua-Wei Yi, Tian Wang, Hui Yang, Meng-Yao Du, Li-Sha Zhu, Xian-Mo Wang, Liang-Cai Xie, Li Wan, Tian Tian

**Affiliations:** 1 Laboratory Department, The First Affiliated Hospital of Yangtze University, Jingzhou, Hubei, China; 2 Hubei Provincial Clinical Research Center for Personalized Cancer Diagnosis and Therapy, The First Affiliated Hospital of Yangtze University, Jingzhou, Hubei, China; 3 Health Management Center, The First Affiliated Hospital of Yangtze University, Jingzhou, Hubei, China; 4 Ophthalmology Department, The First Affiliated Hospital of Yangtze University, Jingzhou, Hubei, China

**Keywords:** antimicrobial stewardship, EUCAST, Gram-negative bloodstream infection, positive blood cultures, rapid antimicrobial susceptibility testing

## Abstract

**Background/Objectives:**

Rapid antimicrobial susceptibility testing (RAST) performed directly from positive blood cultures may facilitate earlier optimization of antimicrobial therapy in bloodstream infections. In this single-center retrospective study, we evaluated the clinical performance of the EUCAST RAST method for Gram-negative bacilli bloodstream infections (GNB BSIs) by assessing its agreement with routine susceptibility testing, its potential to support earlier antimicrobial modification, and its effect on turnaround time (TAT) for susceptibility reporting.

**Methods:**

A total of 229 patients with Gram-negative bacilli detected in positive blood cultures were collected from November 2025 to March 2026. EUCAST RAST was performed directly from positive blood cultures in parallel with the standard antimicrobial susceptibility testing workflow. RAST categorical agreement (CA) with the comparator method, interpretability of results, potential antibiotic modifications based on RAST, and TAT for susceptibility reporting were evaluated.

**Results:**

A total of 187 isolates (81.7%) met the predefined inclusion criteria and were subsequently included in the final analysis. According to the BD Phoenix M50 system, 1.1% of isolates were carbapenem-resistant and 34.3% were extended-spectrum β-lactamase producers. The overall categorical agreement of RAST for all tested antibiotics was 99.4% at 6 h and 100.0% at 16–20 h, with 70% of results interpretable at 6 h. RAST results supported potential antimicrobial modification in 32.1% of patients, including de-escalation in 7.5% and escalation in 24.6%. Among 16 patients receiving ineffective empirical therapy, 13 (81.3%) could potentially have been switched earlier to active treatment based on RAST results. The median TAT for susceptibility reporting decreased from 44 h (IQR, 39.5–47.0) to 26 h (IQR, 21.5–29.0) compared with short-term methods and from 61.5 h (IQR, 58.0–67.0) to 37.5 h (IQR, 35.0–42.0) compared with conventional methods. The overall mortality rate was only 10.7%.

**Conclusion:**

European Committee on Antimicrobial Susceptibility Testing RAST provided reliable early susceptibility results for Gram-negative bacilli directly from positive blood cultures and substantially shortened reporting time, supporting earlier antimicrobial optimization and antimicrobial stewardship.

## 1 Introduction

Due to the high prevalence of antimicrobial resistance among Gram-negative bacilli (GNB), bloodstream infections (BSIs) caused by these organisms are associated with substantial morbidity and mortality (Holmes et al., 2021). In patients with GNB BSIs, ineffective antimicrobial therapy has been associated with mortality rates exceeding 30% (Banerjee et al., 2021; Lee et al., 2012; Moehring et al., 2013; Schwaber and Carmeli, 2007). Globally, sepsis remains a major public health burden: in 2017, an estimated 48.9 million incident cases of sepsis and 11.0 million sepsis-related deaths were reported, accounting for 19.7% of all deaths worldwide (Rudd et al., 2020). Early administration of appropriate antimicrobial therapy has been shown to improve outcomes in patients with GNB BSIs, including shorter hospital stays and lower mortality (Xu et al., 2023). Therefore, rapid organism identification (ID) and antimicrobial susceptibility testing (AST) are essential for timely optimization of antimicrobial therapy and for limiting unnecessary exposure to ineffective broad-spectrum agents.

However, conventional AST methods usually require 24–48 h after a positive blood culture (BC) signal before results become available, which may delay appropriate clinical decision-making. Although molecular diagnostic techniques, such as genotypic testing, enable rapid detection of specific resistance genes, they have important limitations (Peker et al., 2018), including incomplete prediction of phenotypic susceptibility, relatively high cost, and limited availability in many routine laboratories (Yang et al., 2025).

In 2019, the European Committee on Antimicrobial Susceptibility Testing (EUCAST) published a protocol for rapid antimicrobial susceptibility testing (RAST) directly from positive blood cultures using the disk diffusion (DD) method (Jonasson et al., 2020). EUCAST RAST provides interpretive criteria after 4, 6, and 8 h of incubation in the early reading phase. Recent studies have further shown that extending incubation to 16–20 h is feasible and can complement shorter incubation periods when early reading is not achievable or not interpretable (Bianco et al., 2022b; Jonasson et al., 2023). Because it is simple to implement and does not require dedicated additional equipment, EUCAST RAST is well suited to routine clinical microbiology laboratories (Jonasson et al., 2020). Its application may help provide earlier susceptibility information and support antimicrobial stewardship.

In addition, a rapid workflow based on short-term incubation of positive blood culture samples on solid media for 5–7 h, followed by species ID and AST, has been established and routinely used in our laboratory (Ha et al., 2018; Tian et al., 2024). In a previous laboratory evaluation from our center, the overall categorical agreement (CA) for all tested antibiotics were 99.5% at 6 h and 99.7% at 16–20 h when short-term incubation and RAST were incorporated into the workflow (Tian et al., 2025). Our previous studies primarily focused on methodological evaluation, demonstrating the operational feasibility and diagnostic accuracy of RAST. However, the clinical benefits derived from implementing RAST in real-world settings have not yet been assessed. Building on these findings, the present study aimed to clinically evaluate EUCAST RAST in GNB BSIs, with particular emphasis on its agreement with routine AST, its potential to support earlier antimicrobial optimization, and its ability to shorten the turnaround time (TAT) for susceptibility reporting.

## 2 Materials and methods

### 2.1 Study design and clinical samples

This retrospective study was conducted at the First Affiliated Hospital of Yangtze University from November 2025 to March 2026, a 2600-bed university-affiliated medical center in Hubei Province, China. Positive blood cultures incubated in the Bactec FX Automated Blood Culture System (Becton Dickinson, Franklin Lakes, NJ, USA) with gram-negative microorganisms in gram staining were included. Blood cultures showing Gram-positive organisms or polymicrobial growth on Gram staining were excluded. A total of 229 patients with GNB detected in positive blood cultures were collected.

Once flagged positive, blood culture broths were subcultured onto 5% sheep blood agar plates and chocolate agar plates and incubated at 35 °C. Species ID was performed by matrix-assisted laser desorption/ionization time-of-flight mass spectrometry (MALDI-TOF MS; EXS2000, Zybio, Chongqing, China). Routine AST was performed using the BD Phoenix M50 system (Becton Dickinson, USA), which served as the comparator method.

This study complies with the Declaration of Helsinki. The study protocol was approved by Research Ethics Committee of the First Affiliated Hospital of Yangtze University (LL2025-015-01). Informed consent was waived due to the retrospective nature of the study and anonymization of all patient data.

### 2.2 EUCAST rapid antimicrobial susceptibility testing

Rapid antimicrobial susceptibility testing was performed directly from positive blood culture bottles according to the EUCAST RAST guideline (version 7.0). Briefly, 100 μL of undiluted broth from each positive blood culture bottle was inoculated onto a 90-mm Mueller–Hinton agar plate (Guangzhou Detgerm, China) and evenly spread over the plate surface. Antibiotic disks were then applied, and the plates were incubated aerobically at 35 °C.

In our routine laboratory workflow, blood culture bottles flagged positive between 5:00 PM and 10:00 AM were removed and processed within 3 h, and RAST results were read after 6 h of incubation on the same day. For bottles flagged positive after 10:00 AM, RAST was also initiated within 3 h after bottle removal, and the inhibition zones were read after 16–20 h of incubation on the following day. RAST results were interpreted according to EUCAST breakpoints and categorized as susceptible (S), resistant (R), or area of technical uncertainty (ATU), for which no categorical interpretation was assigned.

The following 11 antimicrobial agents were tested using disk diffusion: piperacillin/tazobactam (30/6 μg), cefotaxime (5 μg), ceftazidime (10 μg), ceftazidime-avibactam (10/4 μg), imipenem (10 μg), meropenem (10 μg), ciprofloxacin (5 μg), levofloxacin (5 μg), gentamicin (10 μg), tobramycin (10 μg), and trimethoprim-sulfamethoxazole (1.25/23.75 μg) (Oxoid, UK; Biokont, China).

### 2.3 Comparator AST by short-term incubation and conventional workflow

In our laboratory, different routine workflows were applied according to the time at which blood culture bottles became positive. For bottles flagged positive between 5:00 PM and 10:00 AM, a short-term incubation method was used as previously described (Tian et al., 2024). Subcultures were performed immediately, and after 5–7 h of incubation, colonies grown on blood agar or chocolate agar were identified by MALDI-TOF MS. AST was then performed using the BD Phoenix M50 system with NMIC-413 panels, and results were interpreted according to the current CLSI criteria.

For bottles flagged positive after 10:00 AM, species ID and AST were performed using the conventional overnight culture workflow. Pure colonies grown after overnight incubation were subjected to routine ID and AST using the BD Phoenix M50 system. Extended-spectrum β-lactamase (ESBL) production was determined using the confirmatory testing included in the NMIC-413 panels.

### 2.4 Evaluation of RAST performance

Rapid antimicrobial susceptibility testing results were compared with those obtained by the comparator AST method and classified as CA, very major error (VME), major error (ME), and minor error (mE). CA was defined as concordant categorical results between RAST and the comparator AST. VME was defined as a resistant isolate by the comparator method being categorized as susceptible by RAST. ME was defined as a susceptible isolate by the comparator method being categorized as resistant by RAST. mE was defined as a discrepancy involving intermediate susceptibility in the comparator method and susceptible or resistant categorization in RAST.

Categorical agreement, VME, ME, and mE rates were calculated according to standard definitions. Isolates with RAST results falling within the ATU were considered non-interpretable and were excluded from categorical comparison for that specific drug–organism combination. Standard quality control strains, including *E. coli* ATCC 25922, *E. coli* ATCC 35218, *K. pneumoniae* ATCC 700603, and *P. aeruginosa* ATCC 27853, were used for internal quality control.

### 2.5 Clinical assessment of antimicrobial optimization

The potential clinical impact of RAST was assessed retrospectively. Antimicrobial therapy at the time when RAST results would have become available was reviewed to determine whether treatment could potentially have been modified based on the RAST result. No real-time intervention in patient management was performed.

Appropriate empirical antimicrobial therapy was defined as the use of empirical antibiotics to which the isolate was susceptible according to standard ASTs. Potential antimicrobial optimization was classified as escalation or de-escalation. For this evaluation, antimicrobial agents were grouped as follows (Banerjee et al., 2021): Group 1, gentamicin, tobramycin, ciprofloxacin, levofloxacin, ceftazidime, and other third-generation cephalosporins; Group 2, piperacillin/tazobactam, meropenem, imipenem, ceftazidime-avibactam, tigecycline, and polymyxins.

De-escalation was defined as switching to a narrower-spectrum agent, changing from a Group 2 to a Group 1 agent, or reducing combination therapy to monotherapy. Escalation was defined as switching to a broader-spectrum agent, changing from a Group 1 to a Group 2 agent, or adding one or more antibiotics because of resistance to the empirical regimen.

### 2.6 Turnaround time analysis

Turnaround time was evaluated for RAST and routine AST reporting. TAT comprised two components: (i) the time to positivity, defined as the duration required for microorganism growth in the Bactec blood culture system; and (ii) the processing time, defined as the duration required to generate the final report, including bacterial ID, AST, result validation, and reporting to clinicians.

### 2.7 Mortality

All-cause mortality within 28 days was not the endpoint; rather, we focused on infection-related mortality, defined as death with no other identifiable cause other than the primary infection.

### 2.8 Statistical analysis

Data were analyzed using SPSS version 22.0 (IBM Corp., Armonk, NY, USA). Categorical variables were expressed as numbers and percentages and were compared using the chi-square test or Fisher’s exact test, as appropriate. Continuous variables were presented as median with interquartile range (IQR), depending on data distribution. A two-sided *P*-value < 0.05 was considered statistically significant.

## 3 Results

### 3.1 Study isolates and microbiological characteristics

**FIGURE 1:**
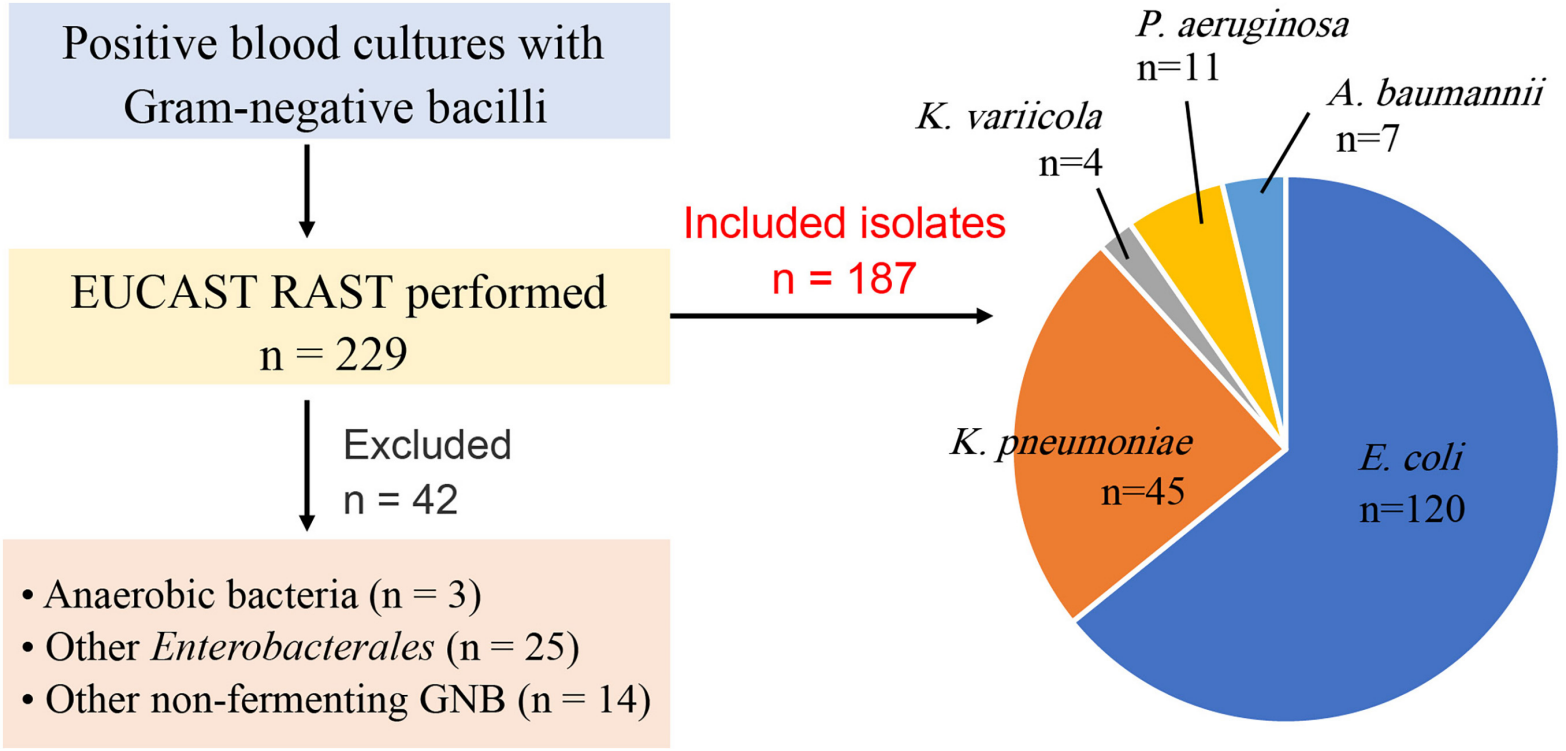
Flow chart of included and excluded Gram-negative bloodstream infection isolates for EUCAST RAST analysis. A total of 229 positive blood culture samples with GNB underwent EUCAST RAST. After exclusion of 42 samples, 187 isolates were included in the final analysis. GNB, Gram-negative bacilli; RAST, rapid antimicrobial susceptibility testing.

During the study period, 229 Gram-negative isolates from positive blood cultures underwent EUCAST RAST, of which 187 isolates (81.7%) met the inclusion criteria and were included in the final analysis. Among these, *E. coli* was the most frequently isolated species (64.2%, 120/187), followed by *K. pneumoniae*/*K. variicola* (26.2%, 49/187), *P. aeruginosa* (5.9%, 11/187), and *A. baumannii* (3.7%, 7/187). A further 3 anaerobes, 25 other Enterobacterales (excluding *E. coli*, *K. pneumoniae*/*K. variicola*), and 14 other non-fermenting bacteria (excluding *P. aeruginosa* and *A. baumannii*) lacked interpretable breakpoints and were excluded (Figure 1).

According to routine AST performed using the BD Phoenix M50 system, 2 isolates (1.1%) were carbapenem-resistant and 58 isolates (34.3%) were identified as ESBL-producing. The distribution of bacterial species and resistance phenotypes is shown in Table 1.

**TABLE 1:** Distribution of Gram-negative isolates and resistance phenotypes identified by routine AST.

Antibiotics	Susceptibility	*E. coli n* = 120 (%)	*K. pneumoniae /K. variicola n* = 49 (%)	*P. aeruginosa n* = 11 (%)	*A. baumannii n* = 7 (%)	Total *n* = 187 (%)
TZP	S	117 (97.5)	46 (93.9)	11 (100.0)	/	174 (96.7)
	R	3 (2.5)	1 (2.0)	0 (0.0)	/	4 (2.2)
CTX	S	69 (57.5)	42 (81.6)	/	/	111 (65.7)
	R	51 (42.5)	7 (18.4)	/	/	58 (34.3)
CAZ	S	95 (79.2)	45 (91.8)	11 (100.0)	/	151 (83.9)
	R	15 (12.5)	4 (8.2)	0 (0.0)	/	19 (10.6)
CZA	S	120 (100.0)	49 (100.0)	11 (100.0)	/	180 (100.0)
	R	0 (0.0)	0 (0.0)	0 (0.0)	/	0 (0.0)
IPM	S	120 (100.0)	49 (100.0)	10 (90.9)	6 (85.7)	185 (98.9)
	R	0 (0)	0 (0.0)	1 (9.1)	1 (14.3)	2 (1.1)
MEM	S	120 (100.0)	49 (100.0)	10 (90.9)	6 (85.7)	185 (98.9)
	R	0 (0)	0 (0.0)	1 (9.1)	1 (14.3)	2 (1.1)
CIP	S	66 (55.0)	42 (81.6)	11 (100.0)	6 (85.7)	125 (66.8)
	R	54 (45.0)	7 (18.4)	0 (0.0)	1 (14.3)	62 (33.2)
LVX	S	67 (55.8)	43 (87.8)	11 (100.0)	7 (100.0)	128 (68.4)
	R	53 (44.2)	6 (12.2)	0 (0.0)	0 (0.0)	59 (31.6)
GEN	S	90 (75.0)	45 (91.8)	/	5 (71.4)	140 (79.5)
	R	30 (25.0)	4 (8.2)	/	2 (28.6)	36 (20.5)
TOB	S	92 (76.7)	45 (91.8)	11 (100.0)	5 (71.4)	153 (81.8)
	R	28 (23.3)	4 (8.2)	0	2 (28.6)	34 (18.2)
SXT	S	67 (55.8)	43 (87.8)	/	5 (71.4)	115 (65.3)
	R	53 (44.2)	6 (12.2)	/	2 (28.6)	61 (34.7)

“/” indicates no data. TZP, piperacillin/tazobactam; CTX, cefotaxime; CAZ, ceftazidime; CZA, ceftazidime-avibactam; IPM, imipenem; MEM, meropenem; CIP, ciprofloxacin; LVX, levofloxacin; GEN, gentamicin; TOB, tobramycin; SXT, trimethoprim-sulfamethoxazole; S, susceptible; R, resistant.

### 3.2 Agreement between EUCAST RAST and routine AST

The performance of EUCAST RAST was assessed by comparison with routine AST results generated by the BD Phoenix M50 system. Overall, RAST showed high agreement with the routine method, with an overall categorical agreement (CA) of 99.4% at 6 h and 100% at 16–20 h (Table 2).

**TABLE 2:** Performance of EUCAST RAST at 6 h and 16–20 h compared with routine antimicrobial susceptibility testing.

Antibiotics	Times (h)	RAST			BD MIC			CA	VME	ME	mE
		R	ATU	S	R	I	S				
TZP	6	3	10	111	4	2	118	98.2	0	0.9	0.9
	16–20	0	1	55	0	0	56	100	0	0	0
CTX	6	40	5	72	44	0	73	100	0	0	0
	16–20	14	0	38	14	0	38	100	0	0	0
CAZ	6	11	19	94	15	9	100	98.1	0	0	1.9
	16–20	2	4	50	4	1	51	100	0	0	0
CZA	6	0	0	124	0	0	124	100	0	0	0
	16–20	0	0	56	0	0	56	100	0	0	0
IPM	6	2	1	125	2	0	127	100	0	0	0
	16–20	0	1	57	0	0	58	100	0	0	0
MEM	6	2	2	125	2	0	127	100	0	0	0
	16–20	0	1	57	0	0	58	100	0	0	0
CIP	6	41	11	77	43	0	86	100	0	0	0
	16–20	18	2	38	19	0	39	100	0	0	0
LVX	6	40	14	75	41	0	88	100	0	0	0
	16–20	17	6	35	18	0	40	100	0	0	0
GEN	6	24	0	98	24	0	98	100	0	0	0
	16–20	12	0	42	12	0	42	100	0	0	0
TOB	6	21	8	100	23	0	106	99.2	0	0.9	0
	16–20	10	2	46	11	0	47	100	0	0	0
SXT	6	45	2	75	47	0	75	98.3	2.1	1.3	0
	16–20	14	0	40	14	0	40	100	0	0	0
Overall	6	229	72	1077	245	11	1122	99.4	0.4	0.3	0.2
	16–20	87	17	514	92	1	525	100	0	0	0

TZP, piperacillin/tazobactam; CTX, cefotaxime; CAZ, ceftazidime; CZA, ceftazidime-avibactam; IPM, imipenem; MEM, meropenem; CIP, ciprofloxacin; LVX, levofloxacin; GEN, gentamicin; TOB, tobramycin; SXT, trimethoprim-sulfamethoxazole; CA, categorical agreement; VME, very major error; ME, major error; mE, minor error; ATU, area of technical uncertainty. Percentages for CA were calculated among interpretable results, excluding ATU results.

At 6 h, MEs were observed for trimethoprim-sulfamethoxazole (SXT, 1.3%) in *K. pneumoniae*, tobramycin (TOB, 0.9%) in one other *K. pneumoniae*, and piperacillin-tazobactam (TZP, 0.9%) in *E. coli*; mEs were observed for ceftazidime (CAZ, 1.9%) in *E. coli* and TZP (0.9%) in *K. pneumoniae*; and VMEs were detected only for trimethoprim-sulfamethoxazole (SXT, 2.1%) in *A. baumannii*. In addition, the proportion of results falling within the area of technical uncertainty (ATU) decreased significantly from 5.3% at 6 h to 2.8% at 16–20 h (*p* < 0.05), indicating improved interpretability with longer incubation.

### 3.3 Patient characteristics

The clinical and demographic characteristics of the included patients are summarized in Table 3. The most common underlying diseases were cardiovascular disease, hypertension, and diabetes mellitus. The major sources of BSIs were the urinary tract (45.5%) and intra-abdominal tract (29.9%), and most cases were hospital-acquired (77.0%).

**TABLE 3:** Clinical characteristics of patients with Gram-negative bacilli bloodstream infections.

Characteristic	Value
Demographics	
No. of patients	187
Age, years, median (IQR)	68 (57–75)
Male sex, *n* (%)	94 (50.3)
Comorbidities, n (%)	
Cardiovascular disease	91 (48.7)
Hypertension	77 (41.2)
Diabetes mellitus	64 (34.2)
Solid cancer	41 (21.9)
Hematological malignancy	6 (3.2)
Chronic kidney disease	25 (13.4)
Chronic liver disease	17 (9.1)
Immunosuppression	9 (4.8)
No comorbidity	18 (9.6)
Source of bloodstream infection, n (%)	
Urinary tract	85 (45.5)
Intra-abdominal tract	56 (29.9)
Primary bloodstream infection	21 (11.2)
Pulmonary infection	12 (6.4)
Vascular catheter-related infection	9 (4.8)
Skin and soft tissue infection	4 (2.1)
Clinical severity, n (%)	
ICU admission	27 (14.4)
Mechanical ventilation	7 (3.7)
Type of acquisition, n (%)	
Community-acquired infection	43 (23.0)
Hospital-acquired infection	144 (77.0)
Laboratory findings, median (IQR)	
Time to blood culture positivity, h	12 (10–14)
White blood cell count, ×10^9^/L	10.2 (7.1–15.3)
Neutrophil count, ×10^9^/L	8.9 (6.1–13.4)
Procalcitonin, ng/mL	4.6 (1.1–13.3)

Continuous variables are presented as median (IQR), and categorical variables are presented as *n* (%). ICU, intensive care unit; IQR, interquartile range.

Inflammatory response was prominent in this cohort, with a median procalcitonin (PCT) level of 4.6 ng/mL.

### 3.4 Potential impact of RAST on antimicrobial therapy optimization and turnaround time

All patients received empirical antimicrobial therapy, and 171 of 187 patients (91.4%) received appropriate empirical treatment. Based on the RAST results available at 6 h and 16–20 h, antimicrobial therapy could potentially have been revised in 32.1% of patients (Table 4), including both escalation and de-escalation. The overall mortality rate was 10.7%. In addition, the mean duration of hospitalization for all patients was 10 d (IQR, 6–14 d).

**TABLE 4:** Impact of EUCAST RAST results on antimicrobial therapy optimization.

Variable	n/N (%)
Empirical antimicrobial therapy	
Appropriate empirical treatment	171/187 (91.4)
Inappropriate empirical treatment	16/187 (8.6)
Potential revision among patients receiving inappropriate empirical treatment	13/16 (81.3)
No potential revision among patients receiving inappropriate empirical treatment	3/16 (18.7)
Potential RAST-guided antibiotic management	
Any potential antibiotic modification	60/187 (32.1)
De-escalation	14/187 (7.5)
Escalation	46/187 (24.6)
Continuing treatment without change	127/187 (67.9)
Clinical outcomes	
Mortality	20/187 (10.7)
Length of hospitalization, days, median (IQR)	10 (6–14)

RAST, rapid antimicrobial susceptibility testing; IQR, interquartile range. Potential antibiotic modification included both escalation and de-escalation based on RAST results. Length of hospitalization was defined as the time from blood culture positivity to discharge. Mortality definition should be kept consistent with the section “2 Materials and methods”.

Rapid antimicrobial susceptibility testing also shortened the TAT for susceptibility reporting (Table 5). When read at 6 h, the median TAT of RAST was 26 h (IQR, 21.5–29 h), compared with 44 h (IQR, 39.5–47 h) for the short-term incubation workflow. When read at 16–20 h, the median TAT was 37.5 h (IQR, 35–42 h), compared with 61.5 h (IQR, 58–67 h) for the conventional workflow.

**TABLE 5:** Turnaround time for antimicrobial susceptibility reporting using EUCAST RAST and routine workflows.

Reading time and organism	No. of isolates	EUCAST RAST TAT, median h (IQR)	Routine workflow TAT, median h (IQR)	Time saved by RAST, h
6 h reading: compared with short-term incubation workflow				
Overall	123	26.0 (21.5–29.0)	44.0 (39.5–47.0)	18.0
*E. coli*	76	25.0 (22.0–28.0)	43.5 (39.8–47.0)	18.5
*K. pneumoniae/ K. variicola*	34	24.5 (20.0–28.0)	43.5 (38.3–47.0)	19.0
*P. aeruginosa*	7	28.0 (27.0–29.5)	46.0 (45.5–47.0)	18.0
*A. baumannii*	6	30.5 (27.5–33.5)	46.5 (45.3–53.0)	16.0
16–20 h reading: compared with conventional overnight workflow				
Overall	64	37.5 (35.0–42.0)	61.5 (58.0–67.0)	24.0
*E. coli*	44	37.0 (35.0–42.0)	61.0 (58.8–66.3)	24.0
*K. pneumoniae/ K. variicola*	15	40.0 (35.0–43.0)	63.0 (57.5–66.5)	23.0
*P. aeruginosa*	4	34.0 (27.5–40.5)	51.5 (41.5–61.8)	17.5
*A. baumannii*	1	50.0	73.0	23.0

TAT, turnaround time; IQR, interquartile range; RAST, rapid antimicrobial susceptibility testing. Time saved was calculated as the difference between the median TAT of the routine workflow and that of EUCAST RAST. IQR was not calculated when only one isolate was available.

## 4 Discussion

In the current era of escalating antimicrobial resistance, delivering timely, targeted, and rational antibiotic therapy has become a critical priority in the management of severe GNB BSIs. In this study, EUCAST RAST showed high agreement with routine AST for GNB directly from positive blood cultures. The technical complexity of broth microdilution (BMD) limits its feasibility in routine clinical microbiology laboratories. Consequently, the commercial BD Phoenix M50 system was adopted as the comparator method to evaluate the concordance of RAST. Notably, given that both RAST and BD systems are ultimately reported in real-world clinical practice, a direct comparison between these two methods yields more intuitive and practical insights for patient management. The overall CA was 99.4% at 6 h and 100% at 16–20 h, which is consistent with previous reports (Cherkaoui et al., 2022; Edmondson et al., 2024; Martins et al., 2020; Messiaen et al., 2025; Özgen Top et al., 2024; Rudd et al., 2020). These findings support the reliability of RAST in our laboratory setting.

The error rates were low overall. Discrepant results were mainly observed for SXT at the 6 h reading, one case of VME was observed in *A. baumannii* and one case of ME in *K. pneumoniae*, suggesting that early interpretation of this agent should be performed with caution. In addition, extended incubation reduced the proportion of results falling within the ATU, which is consistent with previous findings (Tian et al., 2025) and indicates that the 16–20 h reading remains useful when 6 h results are not interpretable. ATU were mostly related to piperacillin/tazobactam, tobramycin, ciprofloxacin, levofloxacin in our study. The RAST method also effectively identified ESBL-producing and carbapenem-resistant bacteria, demonstrating performance comparable to the BD system. Consistent results have also been reported in other studies (Tayşi et al., 2024; Tian et al., 2025).

The main clinical value of RAST in our study was the earlier availability of susceptibility results, with a median reduction of 18 h in reporting time. Based on RAST results, antimicrobial therapy could potentially have been optimized in 32.1% of patients. Previous studies have reported that RAST led to antibiotic revision in over 40% of patients (Cardot Martin et al., 2022; Russell et al., 2019; Strubbe et al., 2023). However, the de-escalation rate was relatively low in our study. This may be explained by the high appropriateness of empirical therapy in our cohort, the frequent use of broad-spectrum agents such as piperacillin/tazobactam and meropenem, and clinicians’ reluctance to narrow therapy early in patients with severe or hospital-acquired infections. Therefore, RAST results alone may not be sufficient to increase de-escalation in routine practice. Consequently, achieving maximal clinical efficacy warrants the prioritization of active, real-time communication of antimicrobial stewardship programs (ASPs) with clinicians subsequent to the reporting of RAST results.

Studies have demonstrated that carbapenem-sparing therapy is a key objective of ASPs, particularly in regions with a high prevalence of ESBL-producing pathogens (Russell et al., 2019). Given the high proportion of ESBL-producing isolates in our hospital (34.3%), the rational use of carbapenems is particularly important. The prevalence of ESBL-producing isolates remains high in China, supporting broader RAST implementation in hospitals to promote judicious carbapenem use.

Meanwhile, RAST enabled significantly faster antibiotic modifications, likely reflecting an earlier transition from empirical to pathogen-directed therapy. TAT of the RAST in this study was 26 h when interpreted at 6 h, and 37.5 h when interpreted at 16–20 h, both significantly shorter than the approximately 72 h required by traditional methods. As a result, the mortality rate in this study was only 10.7%, substantially lower than that reported in previous studies (Rudd et al., 2020). The approach used in this trial provided RAST results that offered optimal information for tailoring antibacterial management of GNB BSIs. Given the retrospective nature of this study without real-time antimicrobial intervention, the observed benefits regarding shorter hospital stays and reduced mortality are preliminary. These findings should be further validated in future prospective studies.

New β-lactams/βlactamase inhibitors, such as ceftazidime-avibactam (CZA), are favored as a first-line anti-infective agent for treating carbapenemase-producing *K. pneumoniae* (CRKP) Infections (Tamma et al., 2024). However, taking in account the increasing isolation of ceftazidime/avibactam-resistant strains (Wang et al., 2020), development of rapid diagnostic tools able to assess susceptibility to CZA is crucial. Notably, even though no strains exhibiting resistance to CZA were detected in our cohort, the RAST assay achieved 100% accuracy for CZA. This finding is in agreement with the results previously reported (Bianco et al., 2022a).

This study has several limitations. It was a retrospective, single-center study with a limited sample size. The absolute number of carbapenem-resistant isolates was low, which limited the ability to draw definitive conclusions regarding the performance of RAST for carbapenem-resistant isolates. We acknowledge that, due to constraints in laboratory and clinical conditions, BMD, which is generally regarded as the reference standard for AST evaluation, was not employed in the present study. Instead, the BD Phoenix M50 system was used as the routine comparator AST method. Therefore, the performance of EUCAST RAST was assessed against a routine automated comparator method rather than the reference standard, which may have affected the accuracy and interpretation of our findings. In addition, interpretable RAST results were not available for all isolates, and the impact on antimicrobial modification was assessed as potential rather than prospectively implemented changes. Further prospective studies are needed to evaluate the clinical value of RAST when combined with real-time antimicrobial stewardship interventions.

## 5 Conclusion

European Committee on Antimicrobial Susceptibility Testing RAST provided reliable susceptibility results for GNB directly from positive blood cultures and significantly shortened the time to reporting. In our cohort, RAST had the potential to support earlier antimicrobial optimization, but its effect on de-escalation may depend on local prescribing practices and antimicrobial stewardship involvement.

## Data Availability

The original contributions presented in this study are included in this article/supplementary material, further inquiries can be directed to the corresponding authors.
